# Selective Feature Generation Method Based on Time Domain Parameters and Correlation Coefficients for Filter-Bank-CSP BCI Systems

**DOI:** 10.3390/s19173769

**Published:** 2019-08-30

**Authors:** Yongkoo Park, Wonzoo Chung

**Affiliations:** Division of Computer and Communications Engineering, Korea University, Seoul 02841, Korea

**Keywords:** brain-computer interfaces (BCIs), motor-imagery (MI), common spatial pattern (CSP), time domain parameters, correlation coefficient

## Abstract

This paper presents a novel motor imagery (MI) classification algorithm using filter-bank common spatial pattern (FBCSP) features based on MI-relevant channel selection. In contrast to existing channel selection methods based on global CSP features, the proposed algorithm utilizes the Fisher ratio of time domain parameters (TDPs) and correlation coefficients: the channel with the highest Fisher ratio of TDPs, named principle channel, is selected and a supporting channel set for the principle channel that consists of highly correlated channels to the principle channel is generated. The proposed algorithm using the FBCSP features generated from the supporting channel set for the principle channel significantly improved the classification performance. The performance of the proposed method was evaluated using BCI Competition III Dataset IVa (18 channels) and BCI Competition IV Dataset I (59 channels).

## 1. Introduction

Brain–computer interfaces (BCIs) enable the translation of neural signals related to a user’s intention into control signals in the absence of muscle movements, and have drawn considerable attention in various research fields, including rehabilitation and engineering [1,2,3]. Due to the technological developments in practicality and portability in recent years, BCIs have also been applied to entertainment and educational filed. [4,5].

Most current BCI systems use electroencephalogram (EEG) extensively due to its high temporal resolution and non-invasiveness [6]. The EEG-based BCI studies show that, when imagining movement of the body, the EEG signals from the regions associated of the cerebral cortex show decreased and increased power in sensorimotor and beta rhythm, called event-related desynchronization (ERD) and event-related synchronization (ERS), respectively [7,8]. Thus, motor imagery (MI)-based BCIs are widely studied by identifying ERD/ERS patterns.

However, EEG signals suffer from low signal-to-noise ratios (SNR) and are highly correlated due to the volume conduction effect [9]. Consequently, they are susceptible to strong artifacts [10,11]. The common spatial pattern (CSP) approach is perhaps the most popular method for extracting ERD/ERS-related features, resolving these difficulties and thus improving the performance of MI-based BCI [12,13,14,15]. The CSP approach designs spatial filters that maximize the variance for one MI-task while simultaneously minimizing the variance for the other task, and extracts discriminative ERD/ERS-related features based on the spatially filtered EEG signals.

Various studies have examined ways to improve CSP algorithms in three categories: frequency optimization, regularization, and channel selection. Frequency range optimization for CSP has been proposed using filter-banks. The filter-bank CSP (FBCSP) approach, described in [16,17], performs the CSP for each frequency band and selects the distinct frequency bands by comparing mutual information of each frequency band CSP features. The FBCSP approach overcomes the frequency range problem of CSP and shows improved performance for MI-classification.

The regularized CSP (R-CSP) [18] approach considers regularization of CSP to overcome the sensitivity thereof to noise and overfitting. However, the performance of R-CSP is highly dependent on optimization of the regularization parameters, which requires exhaustive cross-validation. A frequency range-optimized version of R-CSP, filter-bank regularized CSP (FBRCSP), has also been proposed [19].

Since the CSP approach uses all available channels, including noisy and task-irrelevant channels, the selection of MI-related channels is important for improving the performance of CSP-based algorithms. The sparse CSP (SCSP) approach, described in [20], removes MI-irrelevant channels via sparse CSP filters based on the $\ell_{1}/\ell_{2}$-norm constraint, and applies the CSP to the remaining channels. The CSP-rank for multiple frequency band (CSP-R-MF) approach, described in [21], generates CSP outputs for each frequency band based on the channels with significant conventional CSP filter coefficients, and selects features from the multi-band CSP outputs using the least absolute shrinkage and selection operator (LASSO) algorithm [22]. The experimental results presented in this paper show that these channel selection approaches yield better performance than frequency optimization and regularization.

Although the channel selection CSP approaches improve performance markedly, they have a fundamental limitation that SCSP and CSP-R-MF select MI-relevant channels based on the global CSP, which might already be corrupted by the MI-irrelevant channels. Hence, a CSP-independent method for determining MI-relevant channels is desired.

In this paper, we propose a novel MI-relevant channel selection method for FBCSP. We utilize time domain parameters [23] (TDPs) and correlation coefficients of EEG channel pairs to determine MI-relevant channels. TDPs are extracted from wide frequency band EEG time domain signals and originally used as features to classify MI in [24]. In [24], three types of TDPs are introduced, namely the variance of the signal, the variance of the first derivative, and the variance of the second derivative, which represent activity, mobility, and complexity of the signal, respectively. We consider the channel with the highest Fisher ratio ([25]) of TDPs as the most discriminative channel for MI-tasks and refer to it as the principle channel. We form a supporting channel set for the principle channel with the channels that have high correlation coefficients with the principle channel. Finally, we extract the FBCSP features from the supporting channel set and use them as the input to the support vector machine (SVM) classifier [26]. The performance of the proposed method was evaluated using BCI Competition III Dataset IVa and BCI Competition IV Dataset I. Comparison of the performance with existing CSP-based methods demonstrates significant improvement in classification performance. The rest of this paper is structured as follows. Section 2 presents the proposed method. Section 3 provides the experiment results and discussion. Finally, the conclusion is drawn in Section 4.

## 2. Methods

In this paper, we consider the binary MI-classification. First, let us consider *K* channel EEG signals. We denote the *k*th channel EEG signal at time point *n* as $x^{\left(k\right)}\left(n\right)$, where k=1,2,⋯,K, n=1,2,⋯,N and *N* is the number of time samples per channel. Assume that *I* trials of the MI-EEG signal are available, indexed as $x_{i}^{\left(k\right)}={\left[x_{i}^{\left(k\right)}\left(1\right),x_{i}^{\left(k\right)}\left(2\right),\cdots ,x_{i}^{\left(k\right)}\left(N\right)\right]}^{T}$, where i=1,2,⋯,I. We denote $I_{1}$ and $I_{2}$ as the index set of each MI class ($I_{1}\cup I_{2}=\left\{1,2,\cdots ,N\right\}$).

**Figure 1:**
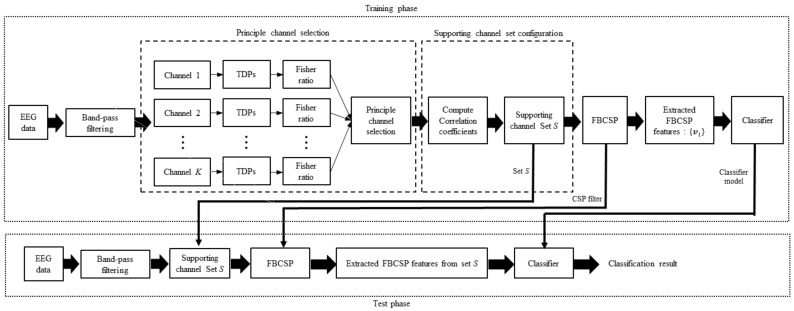
Block diagram of the proposed method.

The block diagram in Figure 1 depicts the proposed algorithm. We first introduce the TDPs, and Fisher ratio of TDPs to measure the MI-relevance of each channel. The channel with the highest Fisher ratio of TDPs is referred to as the principle channel. A supporting channel set for the principle channel is constituted from channels that have correlation coefficients with the principle channel exceeding a certain threshold. The FBCSP features are extracted from the supporting channel set to improve the MI-classification performance.

### 2.1. Principle Channel Selection

The type *p* (p=0,1,2) time domain parameter (TDP) for the *i*th trial of the *k*th channel EEG signal, denoted by $T_{\left(i,p\right)}^{\left(k\right)}$, is defined by the following [23]:

(1) $$\begin{array}{c} T_{\left(i,p\right)}^{\left(k\right)}=\log\left(var\left(\frac{d^{p}x_{i}^{\left(k\right)}\left(n\right)}{dn^{p}}\right)\right),\;\;p=0,1,2. \end{array}$$

The first type (p=0) represents signal power, the second type (p=1) represents the EEG pattern for mean frequency, and the third type (p=2) represents the EEG pattern for frequency change [23].

The Fisher ratio is widely used to measure the class-discriminative property of a parameter by projecting high-dimensional parameters into one-dimensional space [27]. The Fisher ratio of the three types of TDP for channel *k*, defined by $F^{\left(k\right)}$, is given by,

(2) $$\begin{array}{c} F^{\left(k\right)}=\frac{\sum_{p=0}^{2}{\left(\frac{1}{|I_{1}|}\sum_{i\in I_{1}}T_{\left(i,p\right)}^{\left(k\right)}-\frac{1}{|I_{2}|}\sum_{i\in I_{2}}T_{\left(i,p\right)}^{\left(k\right)}\right)}^{2}}{\sum_{p=0}^{2}\sum_{c=1}^{2}\frac{1}{|I_{c}|}\sum_{i\in I_{c}}{\left(T_{\left(i,p\right)}^{\left(k\right)}-\sum_{i\in I_{1}}T_{\left(i,p\right)}^{\left(k\right)}\right)}^{2}}, \end{array}$$

where $|I_{c}|$ denotes the size of the index set $I_{c}$. Postulating that the channel with the highest Fisher ratio has the most significant discrimination between MI tasks, we select the channel with the highest Fisher ratio, denoted by $k_{p}$, and refer to it as the principle channel.

(3) $$\begin{array}{c} k_{p}=\arg\max_{k\in \{1,\cdots ,K\}}\left\{F^{\left(k\right)}\right\}. \end{array}$$

### 2.2. Supporting Channel Set for the Principle Channel

To extract features based on FBCSP, we need sufficient number of channels. For this, we use channels that are highly correlated with the principle channel. The (sample) correlation coefficient between the principle channel $k_{p}$ and a channel *q* for the *i*th trial EEG signals is given by:

(4) $$\begin{array}{c} \rho_{i}^{\left(k_{p},q\right)}=\frac{C\left(x_{i}^{\left(k_{p}\right)},x_{i}^{\left(q\right)}\right)}{\sqrt{P(x_{i}^{\left(k_{p}\right)})}\sqrt{P(x_{i}^{\left(q\right)})}},\;\;q=1,2,\cdots ,K,\mathrm{and}\;q\neq k_{p} \end{array}$$

where

(5) $$\begin{array}{cc} C\left(x_{i}^{\left(k_{p}\right)},x_{i}^{\left(q\right)}\right) & =\sum_{n=1}^{N}\left(x_{i}^{\left(k_{p}\right)}\left(n\right)-\frac{1}{N}\sum_{n=1}^{N}x_{i}^{\left(k_{p}\right)}\left(n\right)\right)\left(x^{\left(q\right)}\left(n\right)-\frac{1}{N}\sum_{n=1}^{N}x_{i}^{\left(q\right)}\left(n\right)\right) \end{array}$$

(6) $$\begin{array}{cc} P(x_{i}^{\left(q\right)}) & =\sum_{n=1}^{N}{\left(x_{i}^{\left(q\right)}\left(n\right)-\frac{1}{N}\sum_{n=1}^{N}x_{i}^{\left(q\right)}\left(n\right)\right)}^{2} \end{array}$$

and the mean correlation coefficient for class *c*, denoted as ${\bar{\rho }}_{c}^{\left(k_{p},q\right)}$, is given by:

(7) $$\begin{array}{c} {\bar{\rho }}_{c}^{\left(k_{p},q\right)}=\frac{1}{|I_{c}|}\sum_{i\in I_{c}}\rho_{i}^{\left(k_{p},q\right)},\;\;c=1,2. \end{array}$$

After calculating the mean correlation coefficients between the principle channel and other channels, we define the supporting channel set for the principle channel, denoted as *S*, as those channels with mean correlation coefficient exceeding a given threshold, $\rho_{thr}$, as follows:

(8) $$\begin{array}{c} S=\left\{q\in \left\{1,2,\cdots ,K\right\}\left|{\bar{\rho }}_{1}^{\left(k_{p},q\right)}\geq \rho_{thr}\;\mathrm{and}\;{\bar{\rho }}_{2}^{\left(k_{p},q\right)}\geq \rho_{thr}\right.\right\} \end{array}$$

### 2.3. FBCSP Applied to the Supporting Channel Set

The FBCSP approach is then applied to the supporting channel set to extract the MI-relevant features. Let us consider the output of the *m*th filter-bank of the supporting channel set *S* at the *i*th trial as $X_{i,m}^{\left(S\right)}$, where m=1,2,⋯,M and *M* is the number of filter-banks. The normalized sample covariance matrix $E_{i,m}^{\left(S\right)}$ is given by:

(9) $$\begin{array}{c} E_{i,m}^{\left(S\right)}=\frac{X_{i,m}^{\left(S\right)}{X_{i,m}^{\left(S\right)}}^{T}}{trace\left(X_{i,m}^{\left(S\right)}{X_{i,m}^{\left(S\right)}}^{T}\right)},\;i=1,2,\cdots ,I, \end{array}$$

where $E_{i,m}^{\left(S\right)}\in R^{\left|S|\times |S\right|}$. Subsequently, the normalized mean sample covariance matrix for the class *c*, denote as ${\bar{E}}_{c,m}^{\left(S\right)}$, is given by:

(10) $$\begin{array}{c} {\bar{E}}_{c,m}^{\left(S\right)}=\frac{1}{|I_{c}|}\sum_{i\in I_{c}}E_{i,m}^{\left(S\right)},\;\;c=1,2. \end{array}$$

Let $p^{\left(m\right)}$ be a spatial filter applied to $X_{i,m}^{\left(S\right)}$. The averaged variance of the spatially filtered EEG signals in *S*, in frequency band *m* for class *c* (∈{1,2}), can be written as ${p^{\left(m\right)}}^{T}{\bar{E}}_{\left(c,m\right)}^{\left(S\right)}p^{\left(m\right)}$. Let $J(p^{\left(m\right)})$ denote the averaged variance ratio between two classes using a spatial filter $p^{\left(m\right)}$,

(11) $$\begin{array}{c} J\left(p^{\left(m\right)}\right)=\frac{{p^{\left(m\right)}}^{T}{\bar{E}}_{\left(1,m\right)}^{\left(S\right)}p^{\left(m\right)}}{{p^{\left(m\right)}}^{T}{\bar{E}}_{\left(2,m\right)}^{\left(S\right)}p^{\left(m\right)}} \end{array}$$

The common spatial pattern (CSP) algorithm [12,13,14] finds the spatial filters that maximize or minimize the averaged variance ratio *J* as denoted by the following equation:

(12) $$\begin{array}{c} \begin{array}{c} p_{max}^{\left(m\right)}=\arg\max_{p^{\left(m\right)}}J\left(p^{\left(m\right)}\right),\;p_{min}^{\left(m\right)}=\arg\min_{p^{\left(m\right)}}J\left(p^{\left(m\right)}\right). \end{array} \end{array}$$

The CSP feature vector, for the *i*th trial data for supporting channel set *S* in the *m*th frequency band, is defined as:

(13) $$\begin{array}{c} v_{i}^{\left(m\right)}={\left[\begin{array}{cc} v_{i,max}^{\left(m\right)} & v_{i,min}^{\left(m\right)} \end{array}\right]}^{T}, \end{array}$$

where

(14) $$\begin{array}{c} \begin{array}{cc} v_{i,max}^{\left(m\right)} & =\log\left(var({p_{max}^{\left(m\right)}}^{T}X_{i,m}^{\left(S\right)})\right),\\ v_{i,min}^{\left(m\right)} & =\log\left(var({p_{min}^{\left(m\right)}}^{T}X_{i,m}^{\left(S\right)})\right). \end{array} \end{array}$$

For each trial *i*, the FBCSP approach selects feature vectors in discriminative frequency bands among the *M* filter-banks by using the mutual information based individual feature (MIBIF) algorithm [17]. The MIBIF algorithm computes the mutual information between feature vectors and its class label to select the discriminative filter-banks. By selecting the best two frequency bands, e.g., $m_{1}$ and $m_{2}$, we obtain the FBCSP feature vector for the *i*th trial data:

(15) $$\begin{array}{c} v_{i}={\left[\begin{array}{cccc} v_{i,max}^{\left(m_{1}\right)} & v_{i,min}^{\left(m_{1}\right)}\; & v_{i,max}^{\left(m_{2}\right)} & v_{i,min}^{\left(m_{2}\right)} \end{array}\right]}^{T}. \end{array}$$

In the training phase, $\left\{v_{i}\right\}$ and their corresponding known class label vector are fed to the SVM classifier.

## 3. Result and Discussion

We evaluated the proposed method in the task of classifying MI-task on publicly available BCI Competition III Dataset IVa and BCI Competition IV Dataset I. The classification performance of the proposed method was compared with TDP [24], FBCSP [17], FBRCSP [19], SCSP [20] and its filter-bank version denoted as FBSCSP, and CSP-R-MF [21].

### 3.1. BCI Competition Dataset IVa

**Figure 2:**
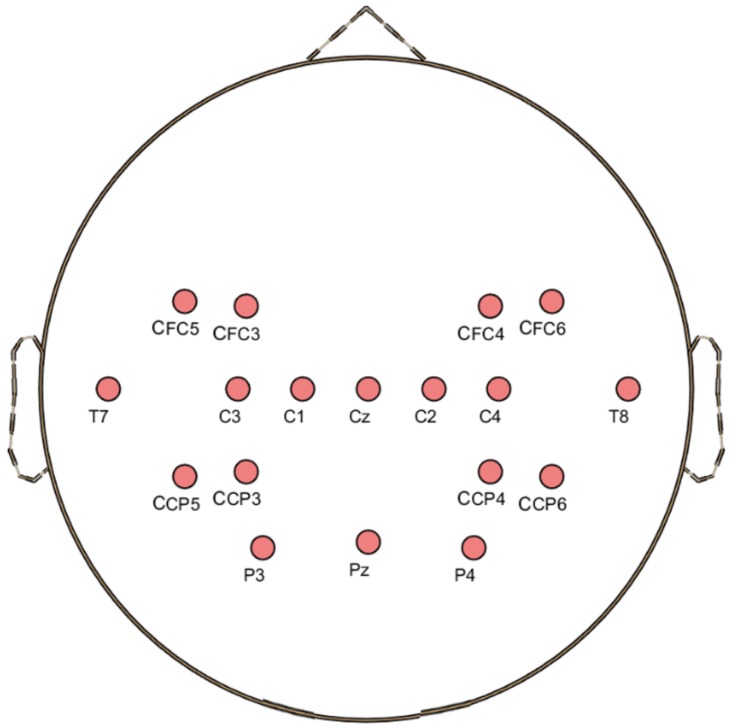
Locations of the 18 channels (K=18) in BCI Competition III Dataset IVa.

The BCI Competition III Dataset IVa [28] was recorded from five healthy subjects, denoted as “al”, “aa”, “av”, “aw”, and “ay”. The five subjects each performed 140 trials involving right hand and right foot MI, divided into training and test set. Table 1 shows the number of training and test sets for the five subjects. Each subject performed the MI over 3.5 s after visual cue, and relaxed for a random length of time (1.75–2.25 s) thereafter. A total of 118 EEG channels corresponding to the positions of the extended international 10/20-system were used for recording, with a sampling rate of 100 Hz. The EEG data were bandpass-filtered between 0.05 and 200 Hz. In this experiment, we selectively used 18 channels (K=18) chosen based on the homunculus theory [29], as shown in Figure 2. The EEG signals in the time segment 0.5–2.5 s after presentation of the visual cue were bandpass-filtered using a fourth-order Butterworth filter operating at 0.5–40 Hz. For FBCSP, eight filter-banks were used for the frequency range 4–36 Hz, dividing evenly at 4-Hz intervals.

### 3.2. BCI Competition IV Dataset I

The BCI Competition IV Dataset I was recorded from four healthy subjects (“a”, “b”, “f” and “g”) and contains two MI EEG signal classes [30]. Fifty-nine EEG channels (K=59) were used to record the data, with a sampling rate of 100 Hz; these were bandpass-filtered between 0.05 Hz and 200 Hz. For each subject, the dataset consisted of 100 trials per class. This experiment used the EEG signals from 0.5 to 2.5 s after cue are used. For TDP extraction, the EEG signals were bandpass-filtered using a fourth-order Butterworth filter operating at 0.5–40 Hz. For FBCSP, eight filter-banks in the frequency range of 4–36 Hz that were divided evenly in 4-Hz intervals were used.

### 3.3. Experiment Results

The threshold, $\rho_{thr}$, used for configuring supporting channel set *S*, plays an important role in classification performance. We determined the optimal threshold value by cross-validation. This can be set as a constant for all subject, or on individual basis for each subject. Although optimization of threshold for each individual subject performed better, this paper presents the experiment results obtained under both settings. Table 2 and Table 3 lists the classification results for the BCI Competition III Dataset IVa. Table 2 shows the classification performance of the CSP approach and its variants, and of the TDP algorithm. Table 3 compares the frequency-optimized CSP variants using filter-banks. The existing frequency-optimized channel selection approaches, FBSCSP and CSP-R-MF, yielded better performance (87.76% and 87.11%, respectively) than the FBCSP and FBRCSP approaches. The proposed method, i.e., frequency-optimized channel selection based on TDP, achieved the best mean classification accuracy, of 89.13%, among all existing algorithms.

Table 4 lists the classification performance in terms of the threshold value ($\rho_{thr}$) and the corresponding size of the supporting channel set (in parenthesis).

Table 5 shows the classification performance of the proposed method for BCI Competition IV Dataset I. In this experiment, we tested the algorithms using a 5×5 cross-validation. The performance of the proposed method was compared with the frequency-optimized channel selection approaches: FBSCSP, the filter-bank version of the sparse CSP (SCSP) [20], and CSP-R-MF [21].

As shown in Table 5, the proposed method yielded the highest mean classification accuracy. Since BCI Competition III Dataset IVa consists of the training and test data specified by BCI Competition III organizers, the performance evaluation based on the Dataset IVa might be overfitted. However, the performance evaluation using BCI Competition IV Dataset I based on cross-validation justified the high performance of the proposed method for an arbitrary training set.

Table 6 lists the classification performance in terms of the threshold value and the corresponding size of the supporting channel set (in parenthesis) for BCI Competition IV Dataset I.

## 4. Conclusions

In this paper, we present a motor imagery (MI) classification algorithm using FBCSP features based on a MI-relevant channel selection. The proposed algorithm uses the Fisher ratio of TDPs and correlation coefficients to obtain a set of channels supporting the principle channel. The FBCSP features generated from the supporting channel set significantly improved the classification performance over existing method, as evaluated using BCI Competition datasets.

## Figures and Tables

**Table 1:** BCI Competition III Dataset IVa.

Subject	Training Data	Test Data
al	224	56
aa	168	112
aw	84	196
av	56	224
ay	28	252

**Table 2:** Classification accuracy of the CSP variations and TDP algorithm for 18 channels (K=18) BCI Competition III Dataset IVa.

Subject	CSP	SCSP	RCSP	TDP
al	94.64	94.6	98.21	100
aa	84.82	88.39	84.82	75
av	61.22	61.22	62.24	65.82
aw	77.68	80.02	81.25	79.46
ay	82.54	82.14	88.49	89.68
mean	80.18	81.28	83.00	82.00

**Table 3:** Classification accuracy of the FBCSP variations and proposed method for 18 channels (K=18) BCI Competition III Dataset IVa.

Subject	FBCSP	FBRCSP	FBSCSP	CSP-R-MF	Proposed Method	Proposed Method
					(Constant Threshold, $\rho_{\mathrm{thr}}=0.6$)	(Individual Threshold)
al	94.64	94.64	100	100	100	100 ($\rho_{thr}=0.6$)
aa	88.39	91.07	90.18	89.29	90.18	91.96 ($\rho_{thr}=0.7$)
av	71.42	75	70.91	73.46	72.45	72.45 ($\rho_{thr}=0.6$)
aw	78.21	76.78	88.39	87.5	88.39	88.39 ($\rho_{thr}=0.6$)
ay	83.73	93.65	89.31	85.31	92.86	92.86 ($\rho_{thr}=0.6$)
mean	83.28	86.23	87.76	87.11	88.78	89.13

**Table 4:** Threshold and classification performance of the proposed method for 18 channels (K=18) BCI Competition III Dataset IVa.

Subject	$\rho_{\mathrm{thr}}=0.6$	$\rho_{\mathrm{thr}}=0.65$	$\rho_{\mathrm{thr}}=0.7$	$\rho_{\mathrm{thr}}=0.75$	$\rho_{\mathrm{thr}}=0.8$
al	100 (13)	98.21 (11)	98.21 (10)	98.21 (7)	98.21 (7)
aa	90.18 (15)	90.18 (12)	91.96 (10)	91.96 (10)	87.5 (9)
av	72.45 (17)	72.45 (17)	70.41(14)	63.78 (12)	58.16 (9)
aw	88.39 (12)	80.80 (10)	77.23 (9)	77.23 (9)	76.34 (8)
ay	92.86 (10)	92.86 (10)	92.86 (10)	87.7 (8)	87.7 (6)
mean	88.78	86.9	86.13	83.78	81.58

**Table 5:** The 5 × 5 cross-validation classification accuracy of the proposed methods and frequency-optimized channel selection methods for BCI Competition IV Dataset I.

Subject	FBCSP	FBSCSP	CSP-R-MF	Proposed Method	Proposed Method
				(Constant Threshold, $\rho_{\mathrm{thr}}=0.75$)	(Individual Threshold)
a	75	79.5	81.5	86.5	**86.5** ($\rho_{thr}=0.75$)
b	54	55.5	63	53.5	57.25 ($\rho_{thr}=0.7$)
f	80.75	82.75	79	89.5	92.5 ($\rho_{thr}=0.8$)
g	92.5	93	87.5	90.5	90.5 ($\rho_{thr}=0.75$)
mean	75.56	77.69	77.75	80.00	81.69

**Table 6:** Threshold and classification performance of proposed method for BCI Competition IV Dataset I.

Subject	$\rho_{\mathrm{thr}}=0.7$	$\rho_{\mathrm{thr}}=0.75$	$\rho_{\mathrm{thr}}=0.8$	$\rho_{\mathrm{thr}}=0.85$	$\rho_{\mathrm{thr}}=0.9$
a	82.75 (27)	86.5 (23)	83.75 (18)	82.5 (12)	82.75 (7)
b	57.25 (49)	53.5 (26)	51.5 (19)	55.5 (12)	55 (8)
f	88.5 (50)	89.5 (43)	92.5 (35)	91.75 (30)	89.5 (17)
g	90.25 (15)	90.5 (15)	83 (10)	82.75 (7)	79.5 (5)
mean	79.69	80.00	77.69	78.13	76.69
